# Association between rat decompression sickness resistance, transthyretin single nucleotide polymorphism, and expression: A pilot study

**DOI:** 10.14814/phy2.16160

**Published:** 2024-07-22

**Authors:** J. Orsat, A. Guernec, C. Le Maréchal, V. Pichereau, F. Guerrero

**Affiliations:** ^1^ Laboratoire ORPHY EA 4324 Univ Brest Brest France; ^2^ Laboratoire de Génétique Moléculaire et d'Histocompatibilité, CHRU Brest, UMR1078 Brest France; ^3^ LEMAR UMR 6539 CNRS/UBO/IRD/Ifremer Univ Brest Brest France

**Keywords:** decompression sickness, rat, transthyretin

## Abstract

Decompression sickness (DCS) is a systemic syndrome that can occur after an environmental pressure reduction. Previously, we showed that the plasmatic tetrameric form of transthyretin (TTR) nearly disappeared in rats suffering DCS but not in asymptomatic ones. In this pilot study, we assessed whether the resistance to DCS could be associated with polymorphism of the gene of TTR. For this study, Sanger sequencing was performed on purified PCR products from the liver of 14‐week‐old male and female standard and DCS‐resistant rats (*n* = 5 per group). Hepatic *TTR* mRNA expression was assessed by RT‐qPCR in 18–19 week‐old male and female standard and resistant rats (*n* = 6 per group). There is a synonymous single nucleotide polymorphism (SNP) on the third base of codon 46 (c.138 C > T). The thymine allele was present in 90% and 100% of males and females standard, respectively. However, this allele is present in only 30% of DCS‐resistant males and females (*p* = 0.0002301). In the liver, there is a significant effect of the resistance to DCS (*p* = 0.043) and sex (*p* = 0.047) on *TTR* expression. Levels of *TTR* mRNA were lower in DCS‐resistant animals. To conclude, DCS resistance might be associated with a SNP and a lower expression of TTR.

## 1 INTRODUCTION

Decompression sickness (DCS) is a systemic syndrome that can occur after a reduction of environmental pressure. It is one of the most dangerous hazards for Self‐Contained Underwater Breathing Apparatus (SCUBA) divers, tunnellers, hyperbaric chamber workers, airplane pilots, and astronauts. This syndrome is characterized by a wide range of symptoms, including articular and muscular pain, skin rashes, and more severe symptoms such as neurological impairments, paralysis, and death (Mitchell et al., 2022). It is acknowledged that the formation of venous gas emboli (VGE) after a reduction of environmental pressure is the triggering factor of DCS. Nevertheless, the development of DCS also depends on physiological mechanisms, which include the nitric oxide‐cGMP pathway (Wisløff et al., 2004), oxidative stress (Mazur et al., 2016), inflammatory response (Bigley et al., 2008; Thom et al., 2015) and activation of the coagulation cascade (Lambrechts et al., 2018; Pontier et al., 2009).

Using a proteomics approach, our group previously reported differences in the plasma proteome of male rats suffering DCS compared to asymptomatic ones after a simulated dive (Lautridou et al., 2016). Among them, the tetrameric form of transthyretin (TTR) nearly disappeared in DCS but not in asymptomatic rats, whereas the plasmatic concentration of the monomeric form of the protein was not modified. Later on, this observation was partially confirmed in divers by showing that TTR was not modified by diving itself (Lautridou, Pichereau, et al., 2017). However, modifications of TTR plasma levels in humans with DCS are still unknown. These data suggested that TTR dissociation could be part of the pathophysiological mechanisms of DCS.

TTR, previously known as pre‐albumin, is a homotetrameric protein of 55 kDa produced mainly by the liver and the choroid plexus but also by many other types of cells including pancreatic islet cells (Wieczorek & Ożyhar, 2021), platelets (Lv et al., 2022) and peripheral blood mononuclear cells (Monu et al., 2021). In the plasma, its principal function is thyroxine (T4) and vitamin A (retinol) transport. TTR is composed of four identical monomers of 127 amino acid residues. Each monomer is made up of eight β‐strands and one α‐helix. The tetramer is formed by monomer–monomer interactions and then dimer–dimer interactions. In some cases, the tetrameric form of TTR can be destabilized, which leads to a tetrameric dissociation. This results in the formation of non‐native monomers and allows the formation of partially unfolded monomeric species (Quintas et al., 2001). These monomers partially unfolded are amyloidogenic precursors responsible of various troubles including inflammation, apoptosis, oxidative stress (Saraiva et al., 2012), or mitochondrial dysfunction, which are also present in DCS. Tetramer dissociation can be favored by low pH, protease‐mediated proteolysis (Peterle et al., 2020), oxidative stress and nitrosylation (Saito et al., 2005), age‐associated protein modifications, or TTR point mutations (Galant et al., 2017). Tetrameric dissociation and the formation of amyloidogenic precursors are also the cause of amyloid diseases. More than 100 TTR point mutations have been reported, and most of them usually lead to a less stable protein than wild‐type TTR (Giampaolo & Vittorio, 2003) and are associated with amyloid diseases (amyloidosismutations.com/mut‐attr.php).

It can therefore be hypothesized that in individuals with less stable TTR variants, the stress induced by diving and decompression might destabilize the tetrameric form of TTR leading to a drop in this form, as already observed in our previous work (Lautridou et al., 2016). If this tetrameric dissociation results in amyloidogenic precursors, they could participate in inflammatory mechanisms, cellular stress, or oxidative stress encountered in DCS. Moreover, this could also explain the well‐known wide interindividual variability for susceptibility to DCS (Berghage et al., 1974; Buzzacott et al., 2014; Lillo & Parker, 2000), whose origin is still poorly understood.

In this pilot study, we sought whether the resistance to DCS could be associated with differences in the coding sequence of the *TTR* gene. Our group previously developed a rat line specifically resistant to DCS, which provides a unique model to decipher the physiological mechanisms leading to DCS (Lautridou et al., 2020; Lautridou, Pichereau, et al., 2017). Hence, we compared (i) the coding DNA sequence of *TTR* and (ii) basal levels of *TTR* mRNA in DCS‐resistant and non‐DCS‐resistant rats.

## 2 MATERIALS AND METHODS

### 2.1 Animal model

In this study, we used male and female rats from the DCS‐resistant strain developed in our laboratory, as previously described by Lautridou, Buzzacott, et al. (2017). Briefly, starting from a founding stock composed of 52 males and 52 females Wistar rats, the DCS‐resistant lineage was obtained thanks to a selective breeding of DCS‐resistant animals after a hyperbaric procedure. To do so, animals were submitted to a standard simulated air dive, then individuals without DCS were selected and bred to create a new generation, subsequently subjected to the same hyperbaric protocol. The prevalence of DCS was 65% for both sexes in animals of the founding stock (first generation) and decreased significantly to 33% in females at the second generation and to 35% in males at the third generation (Lautridou, Buzzacott, et al., 2017). The DCS prevalence subsequently stabilized at 28% and 20%, respectively, for males and females from the 6th generation (Lautridou et al., 2020). Following Generation 6, the selection process continued to maintain the stock and, indeed, the DCS prevalence has remained stable (E. Dugrenot, J. Orsat, & F. Guerrero, unpublished data) which enabled us to consider that the successive generations derive from the same DCS‐resistant strain. Because we aimed to test the relation between the TTR expression and the resistance to DCS at basal state, independently of persistent physiological modifications induced by diving itself (Eftedal et al., 2013; Montcalm‐Smith et al., 2010), a part of the selected individuals were used for our study without being previously exposed to hyperbaric treatment. Selected DCS‐resistant rats were compared to age‐matched standard *Wistar* rats, that is, the same as those we used for the founding stock, obtained from the same breeder (Janvier Labs, St Genêts, France). The standard rats were acclimated to the facility for at least 2 weeks. All animals were housed three per cage under controlled temperature (21 ± 1°C) and lighting (12 h of light per day, 06:00–18:00) at the university animal housing facility until the day of the experiment. They were fed standard rat chow and water ad libitum.

For the sequencing study, we collected the liver of 14‐week‐old standard rats (five females and five males) and resistant rats (five females and five males). To complete the sequencing study, we realized a quantification study. The quantification was conducted on the liver of 18–19‐week‐old standard rats (six females and six males) and resistant rats (six females and six males). To carry out these samples, animals were anesthetized with intraperitoneal administration of Ketamine/Xylazine (80/15 mg/kg, Vibrac, Carros, France) and a lethal blood collection was realized by intracardiac puncture. Livers were then collected and frozen in liquid nitrogen, grounded, and stored at −80°C.

### 2.2 RNA extraction and reverse transcriptase

Total RNA was isolated from rats' liver using the NucleoSpin® RNA Set for NucleoZOL (Macherey Nagel, #740406.50, Hoerdt, France). Briefly, 30 mg of tissue previously ground in liquid nitrogen was homogenized for 2 × 15 s with an Ultraturrax in 500 μL of NucleoZOL (Macherey Nagel, #740404.200, Hoerdt, France). After adding 200 μL of DNase/RNase‐free water and incubating for 15 min at room temperature, samples were centrifuged (12,000 *g*, 15 min, 4°C). 400 μL of the supernatant was collected and the same volume of MX buffer was added. This solution was transferred into NucleoSpin® columns. RNA was fixed at the silica membrane of the columns by centrifugation (8000 *g*, 1 min, room temperature). The silica membranes were then washed and dried with the RA3 buffer containing ethanol. RNA was eluted in 40 μL of DNase/RNase‐free water and stored at −80°C until use. RNA concentrations were measured with a SimpliNano™ spectrophotometer (29‐0617‐12, GE Healthcare Life Sciences) and their purity was assessed using OD_260_/OD_280_ ratios. Their integrity was also checked by electrophoresis on a 1.5% agarose gel with ethidium bromide. 1000 ng of each RNA sample was reverse transcribed with the commercial kit qScript™ cDNA synthesis (Quanta BioSciences, VWR, #733‐1174, France) according to the manufacturer's instruction. The kit contains a reaction mix (dNTPs, oligo(dt) and random primers, enzyme‐specific buffer, and Mg2+) and the Reverse Transcriptase qScript. Complementary DNA (cDNA) was diluted 10‐fold for PCR experiments and stored at −20°C.

### 2.3 PCR for sequencing

PCR experiments were performed with a GeneAmp PCR system 2400 (Applied Biosystems, Thermo Fisher Scientific, France). The amplification of the coding DNA sequence of *TTR* has been done with primers as follows (sense primer, antisense primer, product size): 5′‐ATGGCTTCCCTTCGCCTGTT‐3′, 5′‐TCAGTTCTGGGGGTTACTGAC‐3′, 444 pb (Accession number: NM_012681.2, database: GenBank). 1 μL of 10‐fold diluted cDNA was added to 1X of Phusion HF buffer, 0.2 mM of dNTPs, 0.5 μM of each primer and 0.5 μL of Phusion High‐Fidelity DNA Polymerase (Thermo Fisher Scientific, #F530S, France). The cycling conditions consisted of a denaturing step at 98°C for 30 s, followed by 40 cycles of amplification (denaturation: 98°C for 10 s; annealing: 60°C for 30 s; and extension: 72°C for 30 s). Finally, a final extension was carried out at 72°C for 10 min and samples were held at 4°C. An amplification size control was then performed by electrophoresis on a 1% agarose gel with ethidium bromide. PCR products were then purified with a commercial Kit NucleoSpin® Gel and PCR Clean‐up (Macherey‐Nagel, #740609.50, Hoerdt, France) according to the manufacturer's instruction.

### 2.4 Sequencing

Purified PCR products were sent to Eurofins genomics (Ebersberg, Germany) for Sanger sequencing. For this sequencing, the Light run option was chosen. Sequences were recovered in Serial Cloner software (SerialBasic) and the multiple alignment was done with the website CLUSTAL W (Kyoto University Bioinformatics Center).

### 2.5 Quantification of gene expression by real‐time reverse transcriptase‐PCR (RT‐PCR)

Liver mRNA levels of *TTR* were quantified by RT‐PCR. These experiments were realized with a 7500 Fast Real‐Time PCR system (Applied Biosystems, Thermo Fisher Scientific, France). *TTR* was amplified and quantified by SYBR® green incorporation (EurobioGreen® Mix qPCR 2× Lo‐Rox, #GAEMMX02L‐8 T, Eurobio Ingen) with primers as follows (sense primer, antisense primer, product size): 5′‐TGACAGGATGGCTTCCCTTC‐3′, 5′‐ATCCAGGACTTTGACCATCAGA‐3′, 121 pb (Accession number: NM_012681.2, database: GenBank). The cycling conditions consisted of a denaturing step at 95°C for 2 min, followed by 40 cycles of amplification (denaturation: 95°C for 5 s; annealing/extension step: 60°C for 30 s). Finally, a melting curve program was carried out from 60°C to 95°C with a heating rate of 0.1°C/s, showing a single product with a specific melting temperature for each sample evaluated.

To obtain a standard curve, the *TTR* gene was first amplified from a pool of RT products prepared with all rat samples. PCR products obtained were purified after electrophoretic separation on a 1.5% agarose gel using a commercial Kit NucleoSpin® Gel and PCR Clean‐up (Macherey‐Nagel, #740609.50, Hoerdt, France) according to manufacturer's instruction. PCR products were then quantified using a SimpliNano™ spectrophotometer (29‐0617‐12, GE Healthcare Life Sciences) before proceeding to a serial dilution from 10 ng/μL to 0.01 fg/μL. These ten‐point standard curves were used to determine the PCR efficiency of primers pair and the transcription level in all samples. *TTR* gene was amplified in a single run per tissue from triplicates for standard points and duplicates for sample points. Quantification was normalized using 18S ribosomal RNA (rRNA) as a reference gene. This choice was validated by the absence of significant differences in 18S rRNA levels between experimental groups (*p* > 0.05). All mRNA levels were calculated with the ratio TTR gene mRNA/18S rRNA.

### 2.6 Statistical analysis

All results are expressed as mean ± standard deviation (SD) or median ± interquartile range (IQR). Statistical analyses were performed by using R version 4.2.0 (R Foundation for Statistical Computing, Vienna, Austria). The frequency of the mutation occurrence was analyzed using Fisher's exact test. Normality was tested using Shapiro–Wilk test followed by a Bartlett test or a Levene test. Adapted tests were then performed (ANOVA 2 factors or Kruskal–Wallis test). A *p*‐value <0.05 was considered as significant.

## 3 RESULTS

### 3.1 *TTR* sequencing

Sequencing results were aligned and compared to the *Rattus norvegicus TTR* mRNA (701 bp with 5′ and 3' UTR; Accession number: NM_012681.2, database: GenBank). The sequencing has been processed on the *TTR* coding sequence (444 bp from ATG to Stop). We found a synonymous single nucleotide polymorphism (SNP) at codon 46 (c.138 *C > T* p.Val46=). The frequency of occurrence of these variants is presented in Table 1. Standard rats in both sexes (*n* = 5 each) are homozygous with thymine on position 138 from the coding DNA sequence (CDS), except for one standard male which is heterozygous thymine/cytosine. In DCS‐resistant strain (five males and five females), there are no thymine homozygous rats but there are three rats of each sex heterozygous thymine/cytosine and two rats of each sex homozygous with cytosine on position 138 from CDS. The distribution of these variants' occurrence significantly differs (*p* = 0.00203, Fisher's exact test). The allelic frequency is different between rat strains. The frequency of thymine is 90% and 100% in standard males and females, respectively, whereas this allele is present in only 30% of resistant males and females. There is a significative shift from allele thymine to allele cytosine between standard and DCS‐resistant rats (*p* = 0.0002301, Fisher's exact test).

**TABLE 1:** Frequencies of TTR coding sequence variant (444 bp) in standard and DCS‐resistant rats.

Rat strain	Standard rats		DCS‐resistant rats	
Genotype position c.138	Male (*n* = 5)	Female (*n* = 5)	Male (*n* = 5)	Female (*n* = 5)
*TT*	4	5	0	0
*T/C*	1	0	3	3
*CC*	0	0	2	2
Allele frequency	Cytosine: 5%		Cytosine: 70%	
	Thymine: 95%		Thymine: 30%	
	Cytosine: 10%	Cytosine: 0%	Cytosine: 70%	Cytosine: 70%
	Thymine: 90%	Thymine: 100%	Thymine: 30%	Thymine: 30%

*Note*: A cytosine was substituted by thymine on the codon of Valine 46. Two out of five in each group (DCS‐resistant males (*n* = 5) and females (*n* = 5)) are homozygous for cytosine. The others are heterozygous and none of them are homozygous for thymine. Four out of five standard males (*n* = 5) and five out of five standard females (*n* = 5) are homozygous for thymine. Only one standard male is heterozygous.

### 3.2 *TTR* mRNA quantification

**FIGURE 1:**
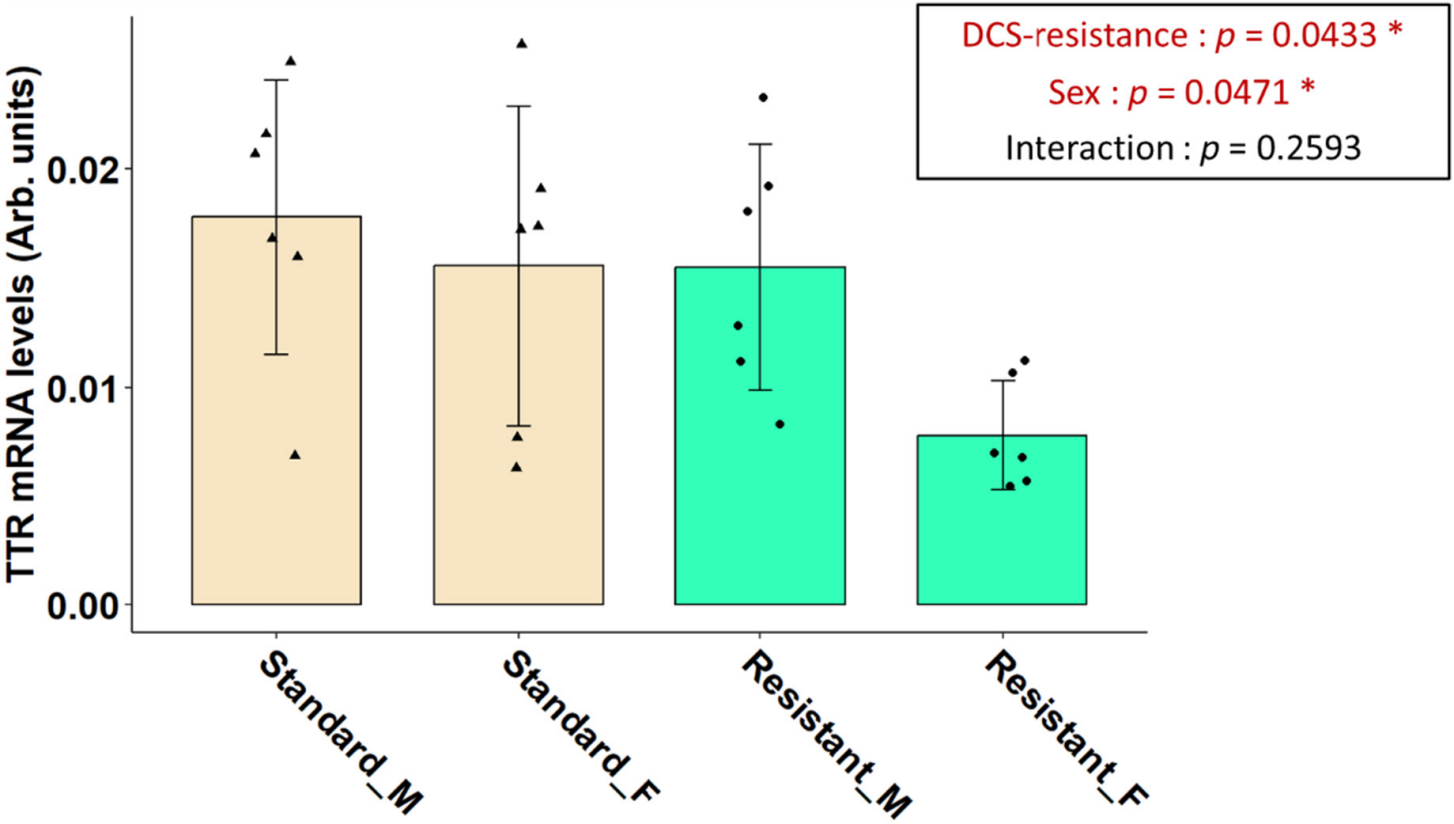
Normalized TTR mRNA levels in the Liver of male (M) and female (F) standard or DCS‐resistant rats. Data are expressed as mean ± SD (*n* = 6 for each group). A *p*‐value lower than 0.05 is considered significantly different.

*TTR* mRNA expression, expressed in arbitrary units (A.U.), was quantified from liver obtained from DCS‐resistant rats and standard rats (Figure 1). These animals have never been subjected to a hyperbaric protocol.

A Shapiro–Wilk test was performed and showed that the data were normally distributed. The ANOVA test showed a significant effect of the resistance to DCS (*p* = 0.0433) and sex (*p* = 0.0471) on *TTR* mRNA expression but no statistically significant interaction between resistance and sex (Figure 1b). *TTR* mRNA expression levels were lower in DCS‐resistant rats (1.164e^−02^ ± 5.778e^−03^ A.U.) compared to standard ones (1.670e^−02^ ± 6.629e^−03^ A.U.). The expression of TTR in the liver was also lower in females (1.169e^−02^ ± 6.624e^−03^ A.U.) than in males (1.665e^−02^ ± 5.829e^−03^ A.U.).

## 4 DISCUSSION

We found in a previous study in rats that the plasma concentration of the tetrameric form of TTR dropped significantly following a DCS, whereas the monomeric form remained unchanged (Lautridou et al., 2016). This drop had not been noticed after a hyperbaric exposure without DCS (Lautridou et al., 2016; Lautridou, Pichereau, et al., 2017). Furthermore, the assessment of TTR plasma levels before the dive didn't show differences between rats that later develop DCS and rats that will be asymptomatic (Lautridou et al., 2016). This led us to hypothesize that the presence of mutations in the TTR coding sequence could lead to the formation of a less stable protein (Yee et al., 2019) and promote the destabilization of TTR during the decompression, leading to DCS.

Therefore, this study aimed to investigate whether a mutation of the TTR gene could account for the occurrence of DCS. For this purpose, we compared the sequence of mature TTR mRNA of two populations of rats which differ by their susceptibility to DCS. Indeed, our group previously developed a rat model of DCS resistance by selectively breeding individuals resistant to DCS from Wistar rats (Lautridou, Buzzacott, et al., 2017). We also showed that, after the same hyperbaric exposure, the occurrence of DCS is 65% in standard Wistar males and females whereas it is 20% for females and 28% for males in the selected population (Lautridou et al., 2020). This DCS occurrence is significantly different and remains stable over generations of selected animals which allows us to state with confidence that the rats used in this study are significantly more resistant to DCS than standard Wistar rats.

In addition, diving elicits acute and chronic effects on the transcriptome of asymptomatic divers (Eftedal et al., 2013), and TTR plasma concentrations are affected by the dive (Domoto et al., 2016) as well as the apparition of DCS (Lautridou et al., 2016). Therefore, DCS‐resistant and standard Wistar rats have never been exposed to a hyperbaric protocol prior to our study. The innovative dimension of our study is to be able to compare these two distinct populations of rats which “naturally” differ in their susceptibility to DCS. This enables us to investigate “pre‐dive” differences possibly linked to the “post‐dive” occurrence (or not) of DCS, which would not have been possible due to the invasive nature of the analyses.

We focused our study on the liver because of its importance in the production of plasmatic TTR but also because of its highly probable implication in DCS (Nyquist et al., 2004). The great majority of the TTR protein present in the plasma is produced by the liver (Vieira & Saraiva, 2014) and, thus, plasmatic TTR concentration is dependent on the expression of the *TTR* gene in the liver. The inhibition of TTR expression specifically in the liver results in a diminution of up to 80%–90% in serum TTR concentration (Adams et al., 2018; Coelho et al., 2013). Moreover, several studies revealed that this organ is also injured by bubbles during decompression (Nyquist et al., 2004; Papadopoulou et al., 2014). In fact, during decompression, one of the main directions for the bubbles appearing into the vein circulation is the inferior vena cava to the liver (Marsh et al., 2023). An elevation of plasmatic markers of liver injury, including alanine transaminase, aspartate transaminase, and lactate dehydrogenase, was reported in mice after a rapid decompression (Peng et al., 2017). They also observed damages in liver histology with disorganized hepatocytes and a disordered lobular structure after the rapid decompression. Histological alterations of the liver were further reported in rats in association with DCS by Mayer et al. (2020) as shown by vacuolization, circulatory disorder, hepatocyte damage and sinusoid dilatation. Taken together, these results indicate that the liver is impacted during the decompression and suggest that it could play a role in the development of DCS.

By analyzing the sequence of mature mRNAs in the liver, we found that the thymine located on the third base of codon 46 in standard Wistar rats is replaced by a cytosine in DCS‐resistant animals (NM_012681.2: c.138C > T). Almost all studies of variants are conducted in humans and available data in rats are therefore relatively limited. Nevertheless, these two variants have been previously described in two different rat strains (Dickson et al., 1985; Duan et al., 1989). Unfortunately, their phenotypes were not compared, and the differences in the physiology between these two variants are still unknown. The replacement of thymine by a cytosine at codon 46 is synonymous, which means that the mutation did not change the sequence of the protein and, at first glance, suggests that the resistance of our strain is not linked to a different TTR protein. Nevertheless, the shift of frequency from thymine to cytosine allele that we observed in our study strikingly paralleled the resistance to DCS. Indeed, male and female standards showed 90% and 100% thymine allele, respectively, while the frequency of the thymine allele dropped to 30% in male and female resistants to DCS. Although the number of animals in the study was small, the difference in frequency was statistically highly significant. As reviewed by Fåhraeus et al. (2015) and Sarkar et al. (2022), numerous human diseases have been reported to be linked to synonymous mutations. These silent mutations can elicit changes in mRNA stability, changes in mRNA secondary structure, disruption of splicing regulation, changes in the pattern of miRNA binding to mRNA, and changes in translational kinetics. Although these mechanisms are poorly understood, silent mutations could affect the structural and functional properties of the protein (Mortazavi et al., 2023). Further, association studies also identified synonymous mutations in genes that do not have a known link to the disease mechanism (Sauna & Kimchi‐Sarfaty, 2011). They are nonetheless clinically important because they can help to predict disease outcomes. Hence, the remarkable distribution of this variant does not totally exclude an effect on DCS, but complementary studies must be conducted before any conclusion can be made.

We also assessed the basal expression of *TTR* and observed a statistically significantly lower basal mRNA level in DCS‐resistant than in non‐DCS‐resistant rats. To our knowledge, the effect of this mutation on the amount of mRNA detected has never been investigated before, and whether it is a consequence of the synonymous mutation cannot be inferred from our study. Our results do not reveal the exact mechanisms underlying the reduced expression of the TTR gene in DCS‐resistant animals. Nevertheless, several other factors have been reported to downregulate TTR gene expression in the liver including hypophysectomy, but not thyroidectomy (Vranckx et al., 1994), administration of Growth Hormone (Vranckx et al., 1994) or peroxisome proliferator clofibrate (Motojima et al., 1992). TTR is also a well‐known negative acute‐phase protein whose gene expression is decreased in response to proinflammatory cytokines (Z. Wang & Burke, 2008, 2010). However, it was shown that the decrease of TTR gene expression, in an acute‐phase response induced by LPS, lasts about 30 h before mRNA levels become higher than in control (Murakami et al., 1988). This suggests that the decreased TTR gene expression in DCS‐resistance is unlikely due to the presence of a low‐level systemic inflammation at the basal state as we hypothesized previously from the increased neutrophil‐to‐lymphocytes ratio (Lautridou et al., 2020).

Chronically lower *TTR* mRNA in the liver has been reported in case of restriction of dietary amino acids (Ingenbleek, 2022), and by exercise training (He et al., 2021). In our study, the average expression of liver *TTR* expression in DCS‐resistant rats is 30.3% lower than in non‐DCS‐resistant. This type of variation was found in mice exercised for 4 weeks where liver *TTR* mRNA was reduced by around 40% which led to about 45% reduction of circulating TTR (He et al., 2021). In another study, a food‐restricted group of rats showed a 27% reduction of liver *TTR* mRNA and approximately a 30% reduction of circulating TTR (le Moullac et al., 1992). In all cases, the decrease in gene expression was associated with an equivalent decrease in plasma protein concentration (He et al., 2021; Ingenbleek, 2022; Militello et al., 2022). We therefore can assume that the plasmatic concentration of TTR is also decreased by around 30% with resistance to DCS. This contrasts with our previous study which didn't show any differences before the dive, between animals that later develop DCS and animals that will not (Lautridou et al., 2016). However, the plasma levels of TTR were assessed by two‐dimensional electrophoresis, and this method may lack of sensibility to show a slight difference between the groups (Lautridou et al., 2016). This remains to be confirmed.

Although less studied, TTR has been shown to interact with metabolism. Indeed, the expression of hepatic TTR is increased by a high‐fat diet and obesity and, at the same time promotes insulin resistance (He et al., 2021). Interestingly, a large genotypic and phenotypic analysis conducted in humans showed an association between low TTR hepatic expression and lower body height (Pathak et al., 2022). Increased TTR expression also increases mitochondrial density whereas its insufficiency triggers a higher degree of oxidative phosphorylation in the liver (Alemi et al., 2021). The lower amount of TTR mRNAs in the liver of DCS‐resistant rats would therefore be coherent with the decrease in body weight observed in DCS‐resistant rats throughout the selection process (Lautridou, Buzzacott, et al., 2017), as well as with the hormonal and energetic remodeling (Vallée et al., 2021) and the lower basal mitochondrial oxygen consumption due to lower mitochondrial volume in skeletal muscle (Lautridou et al., 2020) that we previously reported in DCS‐resistance animals. However, although heavy body weight is a risk factor for DCS, the decrease in body weight contributed statistically significantly to DCS resistance in females only. Conversely, the lower mitochondrial oxygen consumption was detected in males only, no difference was observed between resistant and nonresistant females. The involvement of sex in the mechanisms of resistance to DCS and even in the apparition of this syndrome has been previously suggested. Data from epidemiological studies (Cialoni et al., 2017), as well as animal models of DCS (Buzzacott et al., 2014; Mazur et al., 2014), clearly showed that, for a same dive profile/hyperbaric exposure, females are at higher risk of DCS than males. From these studies and others (Lautridou et al., 2020; Lautridou, Buzzacott, et al., 2017; Vallée et al., 2021), it also appeared that the mechanisms which drive the susceptibility to DCS differ according to sex. Additionally, resistance to DCS was associated with a SNP on the X chromosome (Lautridou et al., 2020). The present study does not show any difference between sexes for the modification of the sequence of the mRNA associated with the resistance to DCS. Although we found that the levels of mRNA are lower in females than in males, the lack of statistically significant sex*resistance interaction indicates that their decrease with DCS resistance is not different between both sexes.

Finally, the classical concentration of TTR in the plasma allows partial inhibition of the production of IL‐1 by monocytes and endothelial cells (Gabay & Kushner, 1999). A decrease in TTR production could lead to higher production of IL‐1 in case of inflammation (Borish et al., 1992). In this way, Desruelle et al. (2019) showed that the inflammatory response capacity could protect mice against DCS. Eftedal et al. (2013) also showed, in the blood of experienced divers without acute hyperbaric exposure, that the expression of genes associated with inflammation, innate immune responses and apoptosis were upregulated as compared to non‐divers. This study highlights that the adaptation to a hyperbaric environment could be done by an enhanced inflammatory capacity and a cellular state of sustained alertness toward exogenous stress. The lower levels of *TTR* mRNA in the liver of DCS‐resistant rats could therefore allow a stronger innate response following decompression stress.

## 5 LIMITATIONS

In this study, we found a lower *TTR* expression in the liver of DCS‐resistant rats but we did not assess TTR concentration in plasma. Given that the liver is the main producer of *TTR*, we therefore considered lower levels of TTR plasma concentration. However, we cannot exclude the possibility that plasmatic concentrations remained unchanged even if most studies showed a close connection between *TTR* hepatic expression and plasmatic concentration. Another limitation is related to our approach which compared selected animals considered resistant to DCS with *Wistar* rats, but none was exposed to hyperbaric procedure. It is not excluded that interindividual variations may bias our data. Moreover, the small sample size reduces the statistical power of our study and is likely preventing us from detecting existing interactions. Finally, although resistance to DCS is associated with differences in both the sequence and the expression of the TTR gene, their involvement in the development of DCS remains to be confirmed and, if so, precise. Indeed, DCS is a multifactorial syndrome involving many confounding factors including hydration status (Wang et al., 2020), age, BMI (Buzzacott et al., 2016), physical exercise before diving or stress (Cialoni et al., 2017), this syndrome cannot be solely explained by genetic factors.

## 6 CONCLUSION

In this study, we found a synonymous SNP between DCS‐resistant and standard rats. Nevertheless, the notable distribution of these variants and the possible impact of the synonymous mutation at different steps require complementary studies. Furthermore, even if this mutation cannot be linked with a known DCS mechanism, it could still represent a predictor helping to assess the susceptibility to DCS. Our study also shows that DCS resistance is associated with a lower basal *TTR* mRNA level in the liver. Given the existing interactions between TTR expression, metabolism and inflammation, this difference could be consistent with the already known mechanisms of DCS as well as the other adaptations previously reported in our model of DCS resistance. Nevertheless, further studies are necessary to support our hypotheses. Whether the modification of TTR observed in rats after a DCS remains to be confirmed in humans.

## AUTHOR CONTRIBUTIONS

J. Orsat, A. Guernec, C. Le Maréchal, V. Pichereau, and F. Guerrero designed the experiment. F. Guerrero and A. Guernec collected the rat samples. J. Orsat and A. Guernec completed all the biochemical analyses. J. Orsat and F. Guerrero wrote the initial draft of the manuscript and all authors contributed, reviewed, and approved the final version of the submitted manuscript. All authors agree to be accountable for all aspects of the work in ensuring that questions related to the accuracy or integrity of any part of the work are appropriately investigated and resolved. All persons designated as authors qualify for authorship, and all those who qualify for authorship are listed.

## FUNDING INFORMATION

We would like to acknowledge the Institut Brestois Santé Agro Matière (IBSAM), Université de Bretagne Occidentale for financial support. The funder had no role in study design, data collection and analysis, decision to publish, or preparation of the manuscript.

## CONFLICT OF INTEREST STATEMENT

The authors have no conflicts of interest to declare.

## ETHICS STATEMENT

Animal experiments were conducted in accordance with the Directive 2010/63/EU of the European Parliament and of the Council on the protection of animals used for scientific purposes, and with the French national laws R214‐87 to R214‐137 of the Rural Code and subsequent modifications. It follows the 3Rs and was approved by the Ethics Committee of the Université de Bretagne Occidentale for Animal Experimentation (Approval no. APAFIS#10395‐2017061909495511).

## Data Availability

The data that support the findings of this study are available from the figshare database (DOI: 10.6084/m9.figshare.22132922; 10.6084/m9.figshare.22132859).
